# The Architecture of a Feasibility Query Portal for Distributed COVID-19 Fast Healthcare Interoperability Resources (FHIR) Patient Data Repositories: Design and Implementation Study

**DOI:** 10.2196/36709

**Published:** 2022-05-25

**Authors:** Julian Gruendner, Noemi Deppenwiese, Michael Folz, Thomas Köhler, Björn Kroll, Hans-Ulrich Prokosch, Lorenz Rosenau, Mathias Rühle, Marc-Anton Scheidl, Christina Schüttler, Brita Sedlmayr, Alexander Twrdik, Alexander Kiel, Raphael W Majeed

**Affiliations:** 1 Chair of Medical Informatics Friedrich-Alexander University Erlangen-Nürnberg Erlangen Germany; 2 Center of Medical Information and Communication Technology University Hospital Erlangen Erlangen Germany; 3 Institute of Medical Informatics Goethe University Frankfurt Frankfurt am Main Germany; 4 Federated Information Systems German Cancer Research Center Heidelberg Germany; 5 IT Center for Clinical Research University of Lübeck Lübeck Germany; 6 Leipzig Research Centre for Civilization Diseases University of Leipzig Leipzig Germany; 7 Institute for Medical Informatics and Biometry Carl Gustav Carus Faculty of Medicine Technische Universität Dresden Dresden Germany; 8 Institute for Medical Informatics University Clinic Rheinisch-Westfälische Technische Hochschule Aachen Aachen Germany; 9 Universities of Giessen and Marburg Lung Center German Centre For Lung Research Justus-Liebig University Giessen Giessen Germany

**Keywords:** federated feasibility queries, FHIR, distributed analysis, feasibility study, HL7 FHIR, FHIR Search, CQL, COVID-19, pandemic, health data, query, patient data, consensus data set, medical informatics, Fast Healthcare Interoperability Resources

## Abstract

**Background:**

An essential step in any medical research project after identifying the research question is to determine if there are sufficient patients available for a study and where to find them. Pursuing digital feasibility queries on available patient data registries has proven to be an excellent way of reusing existing real-world data sources. To support multicentric research, these feasibility queries should be designed and implemented to run across multiple sites and securely access local data. Working across hospitals usually involves working with different data formats and vocabularies. Recently, the Fast Healthcare Interoperability Resources (FHIR) standard was developed by Health Level Seven to address this concern and describe patient data in a standardized format. The Medical Informatics Initiative in Germany has committed to this standard and created data integration centers, which convert existing data into the FHIR format at each hospital. This partially solves the interoperability problem; however, a distributed feasibility query platform for the FHIR standard is still missing.

**Objective:**

This study described the design and implementation of the components involved in creating a cross-hospital feasibility query platform for researchers based on FHIR resources. This effort was part of a large COVID-19 data exchange platform and was designed to be scalable for a broad range of patient data.

**Methods:**

We analyzed and designed the abstract components necessary for a distributed feasibility query. This included a user interface for creating the query, backend with an ontology and terminology service, middleware for query distribution, and FHIR feasibility query execution service.

**Results:**

We implemented the components described in the *Methods* section. The resulting solution was distributed to 33 German university hospitals. The functionality of the comprehensive network infrastructure was demonstrated using a test data set based on the German Corona Consensus Data Set. A performance test using specifically created synthetic data revealed the applicability of our solution to data sets containing millions of FHIR resources. The solution can be easily deployed across hospitals and supports feasibility queries, combining multiple inclusion and exclusion criteria using standard Health Level Seven query languages such as Clinical Quality Language and FHIR Search. Developing a platform based on multiple microservices allowed us to create an extendable platform and support multiple Health Level Seven query languages and middleware components to allow integration with future directions of the Medical Informatics Initiative.

**Conclusions:**

We designed and implemented a feasibility platform for distributed feasibility queries, which works directly on FHIR-formatted data and distributed it across 33 university hospitals in Germany. We showed that developing a feasibility platform directly on the FHIR standard is feasible.

## Introduction

### Context

The COVID-19 pandemic has highlighted the critical need for all countries to strengthen their health data and information systems. Timely, credible, reliable, and actionable data ensure that political decisions are data-driven and facilitate understanding, monitoring, and forecasting [1]. Khan et al [2] have pointed out the need to strengthen national preparedness and the requirement that national public health institutes overcome practical challenges that affect timely access to and use of data. Their analysis identified that the availability of robust information systems that allow relevant data to be collected, shared, and analyzed sufficiently rapidly is needed to provide a timely local response to infectious disease outbreaks in the future [2].

In Germany, the nationally funded Medical Informatics Initiative (MII; funded by the Ministry of Education and Research—Bundesministerium für Bildung und Forschung) through 4 funded consortia (Data Integration for Future Medicine [DIFUTURE] [3], Heidelberg-Göttingen-Hanover Medical Informatics [HiGHmed] [4], Medical Informatics in Research and Care in University Medicine [MIRACUM] [5], and Smart Medical Information Technology for Healthcare [SMITH] [6]) has, in recent years, led to the establishment of data integration centers (DICs) in almost all 34 German university hospitals. These university hospitals created data sharing networks within their respective consortia. However, no overarching cross-consortia research data and feasibility portal existed as of spring 2020.

### Need and Task

To tackle the COVID-19 challenges, the Bundesministerium für Bildung und Forschung has initiated the network of university medicine hospitals, which has launched 13 different projects, for example, to coordinate action plans and diagnostic and therapeutic strategies and to provide a comprehensive COVID-19 data exchange (CODEX) platform [7,8]. Decentralized data collection within the CODEX project was based on the German Corona Consensus Data Set (GECCO), a data set specifically designed to collect data on patients with COVID-19 for research [9].

To make real hospital GECCO data available, university hospitals used Fast Healthcare Interoperability Resources (FHIR) repositories within their MII DIC. To support feasibility studies as part of the German Portal for Medical Research Data (Deutsches Forschungsdatenportal für Gesundheit [FDPG]) and to identify the size of decentral available data sets based on dedicated cohort characterizations (eg, described by Doods et al [10], Soto-Rey et al [11], and Laaksonen et al [12]), we developed a central feasibility portal, securely connected to all German university hospital GECCO FHIR data repositories. For timely design and development, owing to the pandemic, it was imperative to build on tools and experiences from previous projects and align the design for later strategic integration of this feasibility portal into FDPG of the MII [13].

### Background

**Figure 1:**
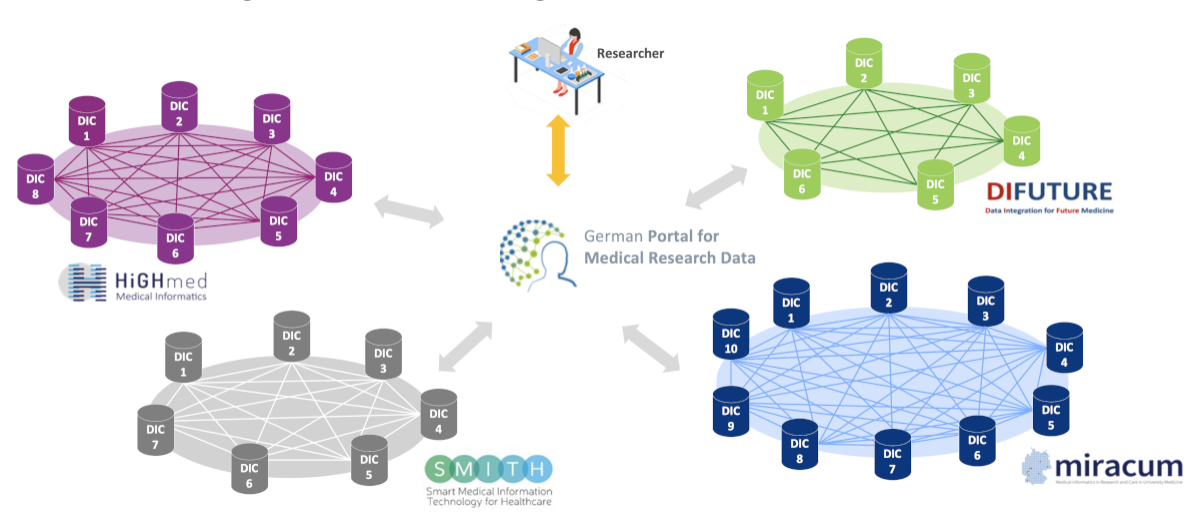
Central German Portal for Medical Research Data and connection to all consortia and data integration centers (DICs; Medical Informatics Initiative).

First, the FDPG shall provide the central access point for researchers (Figure 1) to retrieve information about the availability of routine care data and biosamples in the network of all German university hospitals based on a central feasibility portal (which was, however, not yet developed in 2020). Second, it will provide functionality to electronically apply for data and biosample use in future projects. The latter functionality will manage all incoming research project applications, distribute these electronically to the DICs of all German university hospitals, and keep track of all application status replies from those decentral centers.

To allow studies to query and select patient data from a large, distributed pool of health care institutions, data need to be consolidated across these institutions. In contrast, the hospital landscape is very diverse, with each hospital using different systems and data formats. Although the 4 MII consortia have defined concepts for data harmonization within their consortia DIC (eg, openEHR in HiGHmed [14], the Informatics for Integrating Biology and the Bedside [i2b2] data model [5] in MIRACUM and DIFUTURE, Intersystems HealthShare in SMITH, and the Observational Medical Outcomes Partnership [OMOP] Common Data Model [15,16] in MIRACUM) within the MII, an agreement on a cross-consortia standardized data model was required. Thus, the emerging open standard FHIR [17], developed by Health Level Seven, is a promising candidate for addressing interoperability needs. Health care organizations widely adopt it to achieve interoperability, and it is increasingly supported by major electronic health record vendors. The rapidly increasing availability of data in the FHIR format makes it a natural choice to collect real-world data, while allowing the possibility of translating it to other more specific formats in a relatively simple manner [18]. Thus, the MII working group for interoperability proposed the definition of the MII core data set [19] model based on FHIR. Consistent with this effort, the MII as a whole has agreed on FHIR as the de facto standard for interconsortia communication [20]. Therefore, every DIC in Germany has committed to make its data accessible via the FHIR standard application programming interface (API), making FHIR the only common format supported across all consortia.

### Objectives

The objective of this study was to deduce and illustrate the conceptual design decisions for a distributed feasibility query portal directly based on FHIR data, including the underlying query transformation and execution tools and the middleware components implemented for secure network connections. We also aimed to describe the status of its implementation and use and provide an outlook on its future strategic integration in the German national MII infrastructure.

## Methods

### Abstract Architecture of a Distributed Feasibility Platform

A major challenge for the CODEX project was that any architecture should leverage the power of the German university hospital’s DIC and be compatible with the agreed MII data sharing concepts. Thus, the CODEX project’s feasibility portal was designed to serve as a generic basis for future developments in complementary MII projects. It was further conceived to be extendable to query the MII core data sets.

A feasibility query aims to identify suitable patients for a study. For feasibility, patient privacy can be guaranteed through anonymization by aggregation of the results, while still providing valuable information about the feasibility of a study, as only the number of patients is needed. The task of a distributed feasibility platform is to provide a user with the ability to specify a set of inclusion and exclusion criteria at a central location, send the query to participating sites, translate this query into a search query that can be executed inside a hospital’s research data repository, and return the number of patients matching the criteria combination.

To achieve this, we had to create (1) a user interface (UI; feasibility UI) for creating and managing feasibility queries; (2) a backend service, which translates the user input into a standardized format (Structured Query) using an ontology service; (3) a middleware to securely transport the query; and (4) an execution service, which can process the standardized format, convert it to queries for an FHIR server, and execute the queries. Then, this service should return the number of patients identified.

### Requirement Analysis and Architectural Design

The first step toward developing our tool was to define a list of capabilities (requirements) our platform should support. Building on previous studies on usability [21], query platforms [22], feasibility queries [23], and expert interviews, we curated and prioritized our requirements using Atlassian Confluence as collaboration platform [24]. The prioritization of the features was based on the added value of a feature and the potential estimated implementation cost. The identified features and their prioritization are presented in Multimedia Appendix 1.

The Structured Query as the central part of our feasibility process was developed across multiple meetings with the whole team, including experts on ontology, FHIR, FHIR Search, Clinical Quality Language (CQL), research data repositories, and medical data analysis. From the beginning, it was designed to provide a framework for feasibility queries, which, on the one hand, allowed to create feasibility queries across multiple grouped inclusion and exclusion criteria and, on the other hand, restricted the possible options in a way that makes it easy to translate it into existing FHIR query languages (CQL and FHIR Search). The experts included expertise with existing query tools such as i2b2, OMOP, and Sample Locator [25], previously developed in other projects. This ensured that it would allow for capabilities similar to the existing query tools. The Structured Query, as evidenced by its specification [26], closely resembles the structure of the UI information, while providing sufficient abstraction to separate it from the UI by uniquely identifying single criteria based on their place within a given medical vocabulary. Building on the Structured Query and UI specifications, we worked closely with the whole team to define the necessary UI ontology (UI profiles) and a mapping file for query translation, which was to be used during query translation to enrich the basic definition of a criterion of the Structured Query with query language–specific parameters required for query translation. Critically, by analyzing the CQL language, we found that it has capabilities beyond the requirements of our feasibility specification, and therefore, we would have to specify a subset of CQL for query translation, leading to an incomplete translation, making it more fragile. Therefore, translation from a simpler (specifically restricted) format such as the Structured Query was considered to be easier and allowed us to control further development and separate the representation of the criteria from an implementation-specific system such as CQL and FHIR Search. Furthermore, the Structured Query, although independent of the UI, was designed to resemble it closely, making its generation by the UI easier, as the appropriate query object can be already built by the UI in JavaScript objects, which directly translate to the Structured Query in JSON format. Working across multiple institutions, we also had to consider how the queries and query results are securely exchanged between the different nodes of the network. This was achieved by using middleware components responsible for query transportation. To align the strategy with the other parts of the CODEX project and MII, we evaluated 4 middleware components as part of our project, which had been used previously to transport feasibility queries or used in other parts of the CODEX project to streamline further development. These included the AKTIN broker [27], data sharing framework (DSF) [28], connector component federated search [29], and German Biobank Node Client-Broker [30,31]. We then used the 2 middleware that had the highest scores as a base for further development. To calculate the score, 6 software developers from 5 institutions rated the existing solutions for code quality, documentation, complexity, and suitability for our requirements, on a scale of 1 (very good) to 5 (very bad).

Finally, based on experience from previous studies [22,32] and prototypes for data selection on FHIR servers, we knew that although CQL can support queries involving multiple criteria across different FHIR resources, the capability of FHIR Search is limited. Therefore, if FHIR Search was to be used for more complex queries, a software component was needed to execute and combine single FHIR Search queries to answer more sophisticated feasibility queries.

As part of our project, we performed a usability analysis based on our prototype implementation of the UI, the results of which were fed back into our development process to improve the user experience. This evaluation is described in more detail in a separate publication [33].

**Figure 2:**
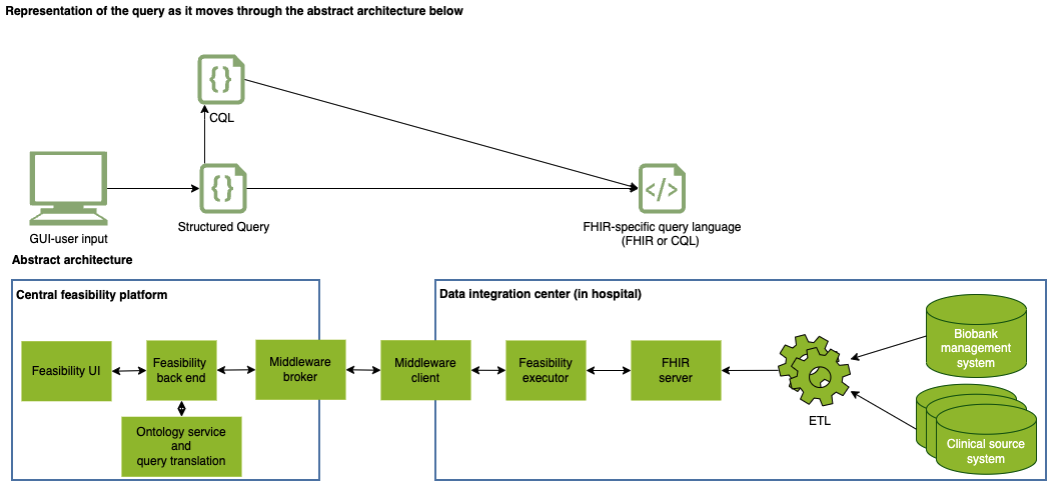
Abstract software components of a distributed feasibility platform. CQL: Clinical Quality Language; ETL: extract-transform-load; FHIR: Fast Healthcare Interoperability Resources; GUI: graphical user interface; UI: user interface.

Figure 2 shows the abstract software components involved in the feasibility process. From left to right, it further illustrates how the representation of the query changes from user input, via a structured representation of the input (Structured Query) to an FHIR query language (FHIR Search [32,34,35] or CQL [16,36-40]) as it moves through the system.

### Performance Analysis

Performance of query execution depends on multiple factors including data set size, type of query execution (CQL vs FHIR Search), query composition (ie, number of criteria within a query), and number of resources processed as part of the query execution.

On the basis of these factors, we created 3 data sets (Table 1) with synthetic FHIR resources that would simulate different server loads and provide data sets, which would return a result for specific queries leading to query-specific data loads from 1, 10, 100, and 1000 thousands of patients. In addition, we augmented 2 of the data sets with background data, which consisted of 413,375 conditions (across 8593 unique condition codes), 270,505 procedures (across 6429 unique procedure codes), and 4,907,600 observations (across 1798 unique observation codes) to represent a typical distribution of data found across a hospital, based on the distributions of a German university hospital. This background data provide data within the server, which are not queried for, but might have an impact on index sizes and query execution speeds.

Furthermore, we created queries that included 4 criteria, each of which would be found exactly 1, 10, 100, or 1000 thousand times. The queries were designed to look for only 1 condition criterion (eg, ICD10–C50.1) or an AND combination of a patient, condition, procedure, and observation criterion (eg, female, ICD10 C50.1, OPS 5-787.ex, and LOINC 55782-7). The combination was always chosen to provide a specific load and has no clinical relevance. They were further chosen to demonstrate a worst-case scenario, where every part would have to be evaluated (AND rather than OR) to provide the answer, as every part would be true for this exact number of patients, implying that the program cannot terminate the search prematurely. We created CQL and Structured Queries for each query and, then, ran all CQL and Structured Queries on the same server 10 times consecutively after 1 warm-up run to ensure the same caching across each query. The host server had 8 cores, 16 GB RAM, and 320 GB solid state drive disk space. The repository for the performance test is available elsewhere [41].

**Table 1:** Performance test data sets.

Data set	Patients, n	Conditions, n	Procedures, n	Observations, n	Overall, n
Small	111,000	111,000	111,000	111,000	444,000
bg^a^-small	111,000	524,375	381,505	5,018,600	6,035,480
bg-large	1,111,000	1,524,375	1,381,505	6,018,600	10,035,480

^a^bg: background.

## Results

### Overview and Implementation

**Figure 3:**
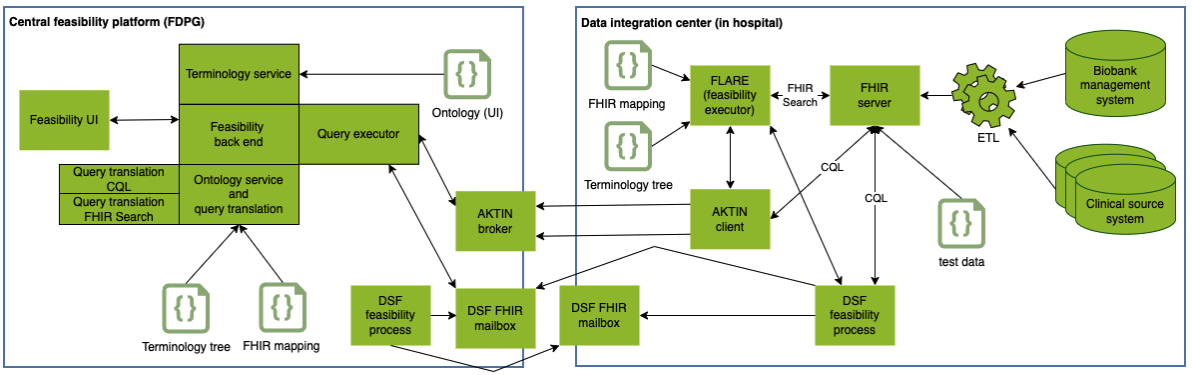
Detailed architecture of the distributed feasibility platform. CQL: Clinical Quality Language; DSF: data sharing framework; ETL: extract-transform-load; FDPG: Deutsches Forschungsdatenportal für Gesundheit; FHIR: Fast Healthcare Interoperability Resources; FLARE: Feasibility Analysis Request Executor; UI: user interface.

While implementing the abstract concept of a feasibility platform explained previously, reusing existing proven software artifacts from previous projects was a major requisite. The proposed architecture ensures strict modularity to achieve flexibility for future extensions and strategic alignments with other developments, for example, in MII. Finally, to fit into the existing architecture designs of the different MII consortia, partial duplication of modules and communication pathways (providing the university hospitals with optional implementation choices) for our development was accepted, when existing modular components could easily be integrated into a coherent framework. The detailed resulting architecture is illustrated in Figure 3.

**Figure 4:**
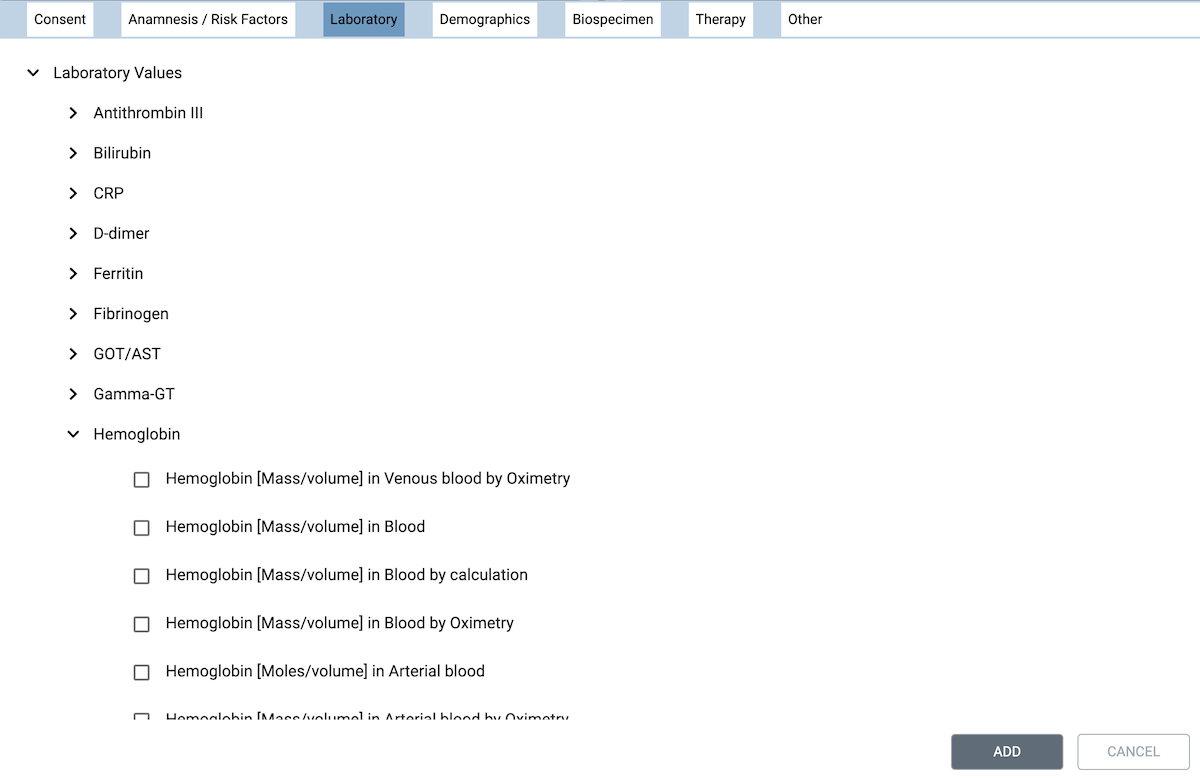
Example user interface representation of an ontology tree.

**Figure 5:**
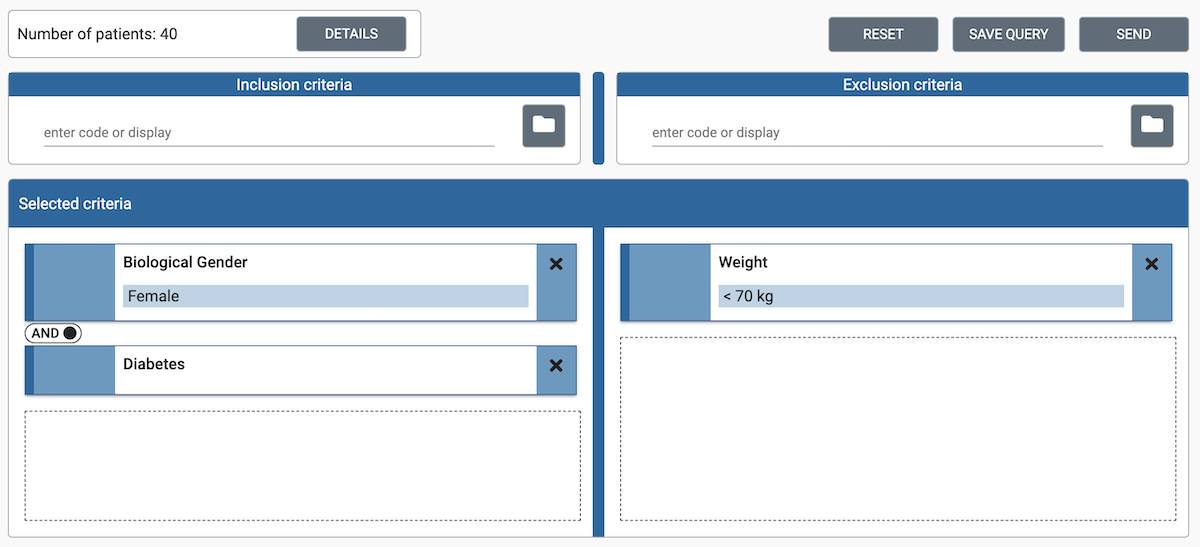
Example feasibility query in the user interface.

The system’s UI (feasibility UI) allows researchers to choose multiple criteria from an ontology tree (Figure 4) and combine them into a set of inclusion and exclusion criteria (Figure 5) using Boolean logic. The inclusion criteria are combined in a conjunctive normal form and the exclusion criteria in a disjunctive normal form.

The ontology (ie, hierarchically structured concepts) for the UI is generated in JSON format based on the underlying FHIR profiles and a terminology service. A detailed description of how the ontology and mapping files are generated is described in a separate publication [42].

The process that generates the UI ontology also generates 2 configuration files (terminology tree and FHIR mapping). These files are required by the central feasibility backend and the decentral FHIR feasibility executor to process the input from the UI and translate it into FHIR-compatible search queries.

Once a researcher has created a feasibility query in the UI, it is converted into our Structured Query format. The Structured Query is a formal representation of the feasibility query, which structures the user input to allow easy translation into different query languages and closely resembles the user input structure. Currently, we support translation into 2 query languages used by FHIR servers: FHIR Search and CQL. Multimedia Appendix 2 illustrates the processing of the Structured Query example shown in Figure 5 from the UI to CQL and FHIR Search.

FHIR Search is part of the FHIR standard and implemented by most FHIR servers. However, currently, complex feasibility queries with intercriterion dependencies are not supported by FHIR Search. A way to overcome this limitation is to break a feasibility query into multiple smaller parts, each of which can be written as a single FHIR Search query. The parts (FHIR Search queries) are then sent to the FHIR server separately. The results are evaluated and combined using set algebra to calculate the final answer for a feasibility query.

For this purpose, we used the software library, Feasibility Analysis Request Executor (FLARE), initially developed for a research project by the University Hospital Rheinisch-Westfälische Technische Hochschule Aachen [43]. For our project, we contributed to the development of FLARE, by extending the software to support the Structured Query.

CQL is a high-level domain-specific query language, which is similar to Structured Query Language, built specifically with medical data in mind [39,40]. It supports, among many other use cases, the definition of cohort characterizations and counting of the respective cohort size, which are needed for feasibility queries. CQL is more powerful than FHIR Search; however, it is not as widely supported by current implementations of FHIR servers. Currently, the popularity of CQL is growing in the FHIR community and has recently been added to the HAPI project [37], a popular open-source FHIR reference implementation. Furthermore, it is supported by the Blaze FHIR server, developed within the German Biobank Alliance project [25], aimed at high-throughput performance. Blaze and the CQL language were chosen as an implementation option in the CODEX project after a comprehensive FHIR server benchmark. CQL has an advantage over FHIR Search in that even complex search queries can be written in a single query, which leads to faster query execution. Therefore, in CODEX, we support both CQL and FHIR Search.

To translate Structured Query to CQL and FHIR Search, we created translation components, which use an FHIR mapping JSON file to map each criterion to its respective FHIR query representation based on its coding (equivalent to FHIR coding type). The information provided by this mapping describes how the criterion is to be searched for inside the FHIR server. This includes the FHIR Search parameters to be used and the type of FHIR resource (eg, observation).

We use a terminology tree JSON file to find all the children of a criterion for inclusion in the respective search. This is necessary as researchers can select groups of criteria by selecting a parent criterion in a terminology hierarchy to include all child elements within the query. An example is the search for the diagnosis *Diabetes mellitus, Type 2*. If a researcher adds the diagnosis *Diabetes mellitus, Type 2* as a criterion (ICD10 code=E11) our tool expands the search to all subtypes of *Diabetes mellitus, Type 2* (including, for example, E11.0—*Diabetes mellitus, Type 2: with coma*). The information necessary to identify all subtypes of type 2 diabetes is provided in the terminology tree file.

A usability analysis of our prototype revealed that it is simple and intuitive. It also showed 26 problems, 8 of which were rated as “critical” [33]. However, usability problems were focused on the presentation of the UI or the ontology and will have no impact on the architectural decisions made. Specifically, our architecture will allow us to resolve these problems independent of the rest of the system for query translation, transportation, and execution.

### Supporting Multiple Query Paths

CQL and FHIR Search have slightly different requirements regarding query execution. We generate multiple representations of the same feasibility query in the central feasibility backend and send all of them to each decentral DIC. This allows the DIC to configure which query representation to use without changing the central implementation. All feasibility queries can be generated as a single CQL query. Therefore, we generate the CQL query centrally and send this query to the DICs and their FHIR servers, which can execute them directly. As, in most cases, the FHIR Search representation of a Structured Query cannot be generated as a single query, we send the Structured Query to the DIC. Each DIC that prefers to use FHIR Search for the query execution will use the FLARE component locally. It translates each Structured Query into FHIR Search queries inside the respective DIC using the mapping and terminology tree files and executes them against the FHIR server.

### Supporting Multiple Middleware

In our architecture, a middleware has the task to securely transport the query into a DIC and transport the answer to a query back to the central platform. In our case, the query is an object that contains the serialized version of our different query representations—Structured Query and CQL. To secure the connection between our central middleware components and local middleware clients, without requiring the university hospitals to open their firewall for outside requests, we chose a pull transport mechanism instead of a push process from the outside. Within CODEX, we evaluated multiple middleware components already developed by various MII partner sites and chose 2 that are already widely used in different consortia and fulfill the requirements mentioned previously: AKTIN broker [27,44] and HiGHmed DSF [28]. Both were extended to fully comply with the CODEX requirements, leading to a new client release for AKTIN [45] and the creation of a feasibility process for the DSF [46], similar to that created by Wettstein et al [47].

### Privacy Through Anonymization by Aggregation and Access Restriction

The system we present here allows for querying patient data across multiple institutions from a central location, and information about patients is leaving the respective institution. This information, as any information about patients, is sensitive and needs to be anonymous when leaving an institution. The nature of feasibility queries is such that only an integer number leaves each participating hospital. However, a potential reconstruction of a patient profile by the central location would be possible if the exact number was returned. To avoid this, we aggregate each result by rounding it to the nearest 10 patients. A result of zero is returned as zero. We further restrict access to the platform to registered users and track all the created feasibility queries.

### Containerization and Deployment Across Hospitals

The system described here is composed of many connected pieces of software, which must be installed across many institutions to create a feasibility query network of participating institutions. To ensure easy distribution of the software and streamline the installation process, we ensured that each software component created is distributed as a Docker image. We further tested our implementation for Kubernetes and installed a version of it in the Kubernetes cluster of the DIC of the university hospital in Erlangen, Germany. For easy installation across the institutions, we created an installation package, which provides an easy-to-install package based on multiple docker-compose files. In this first installation, the sites used only the AKTIN middleware, as the DSF was still in development and the set-up of the DSF proved to be more complex; for example, specific client certificates issued by an official certificate authority were required for its use. During the installation process, we found that the sites had very stringent firewalls and needed the option to support a proxy server between the client inside the hospital and the central broker. After adding proxy support, all the participating institutions could install the software and join the feasibility network.

### First Ontology Generation and Test Across Hospitals

We implemented the architecture described previously and generated an ontology, a terminology tree, and a mapping file based on the GECCO FHIR profiles. We then distributed our implementation across the 33 participating institutions and asked them to load synthetic test data into their respective FHIR servers. We deployed the central feasibility tool and sent queries across the institutions. The test data set was generated based on synthetic data and converted to the MII FHIR format. We then used our UI to generate multiple test queries and found that we could create and execute them on our chosen FHIR servers. We further created a synthetic test patient data set in FHIR format [48] using the electronic data capture tool, REDCap (Research Electronic Data Capture; Vanderbilt University) [49], used by many participating institutions to capture COVID-19 data. The data set contains each type of criterion available in our UI. We verified our implementation using this data set.

### Performance and Query Execution Speed

By running the performance tests (Table 2), we found that CQL was faster than FLARE as the number of resources processed increased. We also found that query execution time increased with the number of resources processed for a search and the amount of background data. For queries where small result sets had to be processed (<100,000 resources) and large amount of background data were loaded into the server, FLARE was faster than CQL. CQL processed all requests in <30 seconds. FLARE did not perform well with very large data sets and queries where >1,000,000 resources had to be processed, leading to execution times >47 seconds.

**Table 2:** Query response times across data set, query, and query execution type (CQL^a^ and FLARE^b^).

Query	Criteria search for	Patients found, n	Resources processed, n	Response time by query execution and data set type (seconds), mean (SD) of 10 consecutive runs					
				cql-small	flare-small	cql-bg^c^-small	flare-bg-small	cql-bg-large	flare-bg-large
0	4	0	0	0.22 (0.01)	0.03 (0.0)	0.3 (0.01)	0.04 (0.0)	1.56 (0.04)	0.04 (0.0)
1000-1	1	1000	1000	0.57 (0.09)	0.11 (0.0)	0.85 (0.19)	0.11 (0.01)	5.52 (0.54)	0.13 (0.01)
1000-all	4	1000	4000	0.25 (0.03)	0.23 (0.06)	0.35 (0.05)	0.24 (0.06)	1.82 (0.06)	0.37 (0.26)
10000-1	1	10,000	10,000	0.56 (0.08)	0.5 (0.01)	0.89 (0.08)	0.49 (0.01)	5.49 (0.21)	0.67 (0.07)
10000-all	4	10,000	40,000	0.35 (0.04)	0.99 (0.08)	0.68 (0.07)	1.0 (0.1)	2.1 (0.08)	1.94 (1.37)
100000-1	1	100,000	100,000	0.85 (0.11)	4.34 (0.07)	1.13 (0.09)	5.16 (0.21)	6.07 (0.26)	5.37 (1.18)
100000-all	4	100,000	400,000	1.48 (0.12)	8.25 (0.41)	2.65 (0.23)	9.65 (0.24)	4.16 (0.18)	10.8 (0.09)
1000000-1	1	1,000,000	1,000,000	N/A^d^	N/A	N/A	N/A	10.49 (1.26)	47.53 (1.35)
1000000-all	4	1,000,000	4,000,000	N/A	N/A	N/A	N/A	29.05 (2.38)	119.64 (4.51)

^a^CQL: Clinical Quality Language.

^b^FLARE: Feasibility Analysis Request Executor.

^c^bg: background.

^d^N/A: not applicable.

## Discussion

### Principal Findings

We presented the concept and implementation of a distributed feasibility query platform, which works directly with FHIR-formatted hospital data. This demonstrates that the FHIR standard is suitable to build a feasibility platform on. FHIR Search does not support feasibility queries across multiple criteria directly. However, we built an FHIR feasibility executor, which combines single queries to answer these feasibility queries. This executor needs to load and combine the results of the different subqueries and, therefore, will be a performance bottleneck if single queries return large data sets. Therefore, we also offer the fast but less widely available CQL query option. Furthermore, separating the concerns and supporting multiple query languages for query executions allows us to adjust to individual institutions’ needs. Similarly, we found it to be useful to support multiple middleware components by providing clear interfaces. This supports more organizations and strategic directions and allows focusing on one middleware (AKTIN), while the other (DSF) is still being developed and deployed. Comparing the 2 middleware, the AKTIN implementation has the advantage of being simple, which is easy to maintain and extend for the purpose of transporting feasibility queries. It is agnostic to the query transported, so that the process extension necessary for the AKTIN implementation was easy and fast to achieve. Furthermore, the AKTIN middleware has been used successfully for several years in other projects. The DSF is an FHIR-based middleware and focuses on providing a platform for defining processes, which can be run across institutions. This enforces more structure than the AKTIN middleware, in the hope that this leads to improved interoperability. The DSF allows peer-to-peer communication if required. However, peer-to-peer communication is not relevant for feasibility queries from a central location. The biggest disadvantage of the DSF is that, with its large feature set, structure, and interoperability, it also introduces a high complexity to the system. Furthermore, the DSF is still in development and is yet to be used in a production environment. We chose to support both middleware in this project, as both have advantages and disadvantages, and the use of either within our future architecture largely depends on their respective use and acceptance within the MII.

Centering around the newly defined Structured Query format, which formally describes a feasibility query, allows the separation of the UI ontology from the translation into FHIR-compatible query languages. Therefore, the platform is built in a modular fashion and highly extendable. For example, one could imagine that an entirely different UI could be developed and integrated into the platform to satisfy future requirements, as long as it creates a Structured Query. Similarly, it allows the ontology, mapping, and what query execution languages the Structured Query is translated into, to be changed, to work with future query languages (eg, if the scope of the underlying data set changes). This allows the ontology for the front end to be created completely independent of the mapping and does not require a specific format for an ontology, allowing for quicker ontology generation compared with approaches that extend existing research platforms such as i2b2 [35]. The Structured Query can be considered as a new internal format for feasibility queries, and it could be argued that the representation as a Structured Query is not as interoperable as an FHIR representation. However, given the need to translate the query into multiple languages before being sent across institutions and that the Structured Query closely resembles the user input, the conversion from user input to Structured Query is much simpler than generating an analogous FHIR representation, which would then be converted again to FHIR Search and CQL. Furthermore, currently, no FHIR specification for feasibility queries exists, which would match the complexity of our Structured Query [32].

In the proposed architecture, the ontology and mapping to FHIR are added using the generated files. Thus, the used ontology and mapping to FHIR can be easily changed. This allows the feasibility platform to extend beyond our project and national boundaries. It is important to consider that any ontology used must be agreed by the institutions participating in a data sharing network and either be applicable directly or mapped at the decentral location according to the rules set by the institution. The FHIR standards’ wide applicability, its wealth of complexity, and medical data entities it can support makes this a feasibility tool that can work with very diverse data, from laboratory data to conditions or biological specimen data. The translation and mapping we created is not restricted to a few FHIR resources, and the platform allows for the extension of the ontology and mapping to any FHIR resource. The fact that we generate mapping files, which can be distributed with our software, meant that the participating sites do not have to open an extra connection to a central terminology server or provide a terminology server themselves. This increases security and ease of installation.

### Related Work

The FHIR standard has become more popular in recent years. More recently, it has been investigated not only for the exchange of patient data but also as a tool for data selection, extraction, and analysis [22,35]. With the popularity of the standard and the MII deciding to use FHIR as its main format for data exchange [19], the task was to build tools directly on the FHIR standard, rather than transforming data further to be analyzed with other software such as OMOP and i2b2 and tools built on their data models, such as Shared Health Research Information Network [50]. In this study, we designed and implemented a feasibility tool, which clearly separates the concerns of the different components and defines clear interfaces. This makes it easy to extend the platform and exchange components at each step of the process from user input to query execution and data storage. Similar to Paris et al [35], we present a feasibility platform, which works directly with the FHIR standard. Unlike Paris et al [35] we present a distributed system, which not only supports the translation of a query to FHIR Search but also the more powerful CQL query language. Hereby, we pave the way for translating standardized feasibility queries into other query languages based on structured input query, mapping, and term-code tree to resolve ontology hierarchies. Our implementation has the distinct advantage of allowing us to map user input to FHIR directly, rather than mapping user input to i2b2 objects and, then, to FHIR, thus reducing the overall complexity. Finally, as the usability of the existing feasibility UIs of i2b2 and OMOP can still be improved [21], the current architecture included and implemented a new and modern UI, which was found in our usability analysis to be intuitive and easy to use. Furthermore, the Sample Locator [25], previously developed as part of the German Biobank Alliance (originating from previous work in the German Cancer Consortium [51]) had the following limitations. (1) It did not include a generic terminology-based ontology tree, which allows researchers to select concepts easily. The current selection criteria were hard-coded, thus hindering the flexible extension of the UI. This is especially important, as the scope of the project will grow over time. (2) It did not allow for more complex queries, such as grouping different criteria in OR groups within AND groups. Therefore, conjunctive (inclusion criteria) and disjunctive (exclusion criteria) normal form was chosen for the new UI, which supports more complex queries. (3) It allowed for direct *not* exclusion of criteria, which is currently not supported for all FHIR Search queries and would have introduced more complexity into the query translation process and had implications for performance (an FHIR Search for*: not* condition C50.1 would have returned a set of all other conditions). (4) It did not support time restrictions across all criteria.

The system’s modular design supports different software components and final architecture decisions within the various MII university hospitals, depending on their local architecture design already existing within their DICs. Modularity with clearly defined APIs means that the comprehensive architecture framework can be adjusted easily, with locally preferred microservice components, if they fulfill the same functionality, thus supporting varying local requirements.

Beyond the analysis of the systems, as part of this study, there are many competing infrastructures for standardizing and distributing queries in a privacy-preserving manner. Specifically, for distributed analysis, multiple frameworks such as the Personal Health Train (PHT) [52,53] and DataSHIELD [54,55] exist. However, here, the focus is on a distributed feasibility platform for standardized feasibility queries that preserves privacy by aggregation at each site. This makes infrastructures such as the PHT and DataSHIELD, which focus on interactive and custom analyses, less well suited for our purpose. PHT specifically focuses on distributing custom analyses (algorithm+query) using containers to move the algorithms to the data. This is a great strength of the PHT, but it is not applicable for a structured feasibility query, which can be executed in the exact same manner (by the same algorithm) every time. Currently, the feasibility platform does not provide a mechanism for multiparty computing, allowing for exact responses to privacy-preserving feasibility queries across sites. This might be potentially relevant for rare diseases, where low numbers of patients would otherwise be returned to each site, thus making more accurate numbers essential. Previous work such as the PHT or DSF could be extended to provide a multiparty computing approach to return exact feasibility answers aggregated across multiple institutions. In the system described here, only the middleware would have to be replaced or extended, as the UI, query generation, and query execution at the sites would be identical.

### Limitations

A feasibility platform across institutions works only if the institutions agree on the same ontology and map their data to the same terminologies or provide a mapping from the given input to their terminologies. In our project, we built on the German DIC data harmonization efforts. This ensures the compatibility of our queries with the data in each participating institution, as all DICs convert the data according to the same FHIR profiles and implementation guides of the MII core data set [56] and the GECCO [9] data set. Not all countries have these DICs, which means that extra data harmonization efforts would be required, which can be expensive and time-consuming. Furthermore, many electronic health record providers now support FHIR, but this does not necessarily mean that they provide the consented profiles or terminologies necessary for a distributed query. Creating a good ontology that is easy to use and provides the researcher with the right criteria is a difficult task. Many institutions generate ontologies manually, which means that they are carefully curated, but this is expensive and time-consuming. We successfully generated an ontology and mapping in an automatic process based on FHIR profiles and an ontology server. Whether this is applicable to arbitrary FHIR profiles still needs to be investigated.

The way we implemented the FHIR Search query path for multicriteria grouped feasibility queries means that the result sets of the sub-FHIR Search queries must be downloaded, patient IDs must be extracted, and the resulting sets must be combined. This download process may not be feasible for queries where parts return many results. To address this problem, currently, we also support CQL, which is a better option for large data sets. Our performance test demonstrated that CQL answers queries processing multiple millions of resources within 30 seconds. FLARE answered queries where 400,000 resources had to be processed in <12 seconds. Specifically, for COVID-19 data sets, we currently do not expect 1 site to return millions of patients, which means that the current implementation will answer queries on patients who are specific to COVID-19 in seconds rather than minutes. Furthermore, the finding that the number of resources processed is the main predictor of query execution time paves the way for future improvements. The current performance test, as well as being repeatable, allows one to draw conclusions on feasible data set sizes. However, a more comprehensive investigation with data sets of 200 or 500 million resources and different server sizes and better understanding of what large real-world data sets look like are still missing. This is especially relevant within the MII if the current feasibility portal is extended beyond the COVID-19 data set to analyze multiple years of real-world hospital data.

### Future Directions and Conclusions

We presented the design and implementation of a feasibility platform for distributed feasibility queries, which works directly on FHIR-formatted data. The platform was deployed across 33 university hospitals and the viability of the approach was demonstrated using a set of synthetic test data in the appropriate format. Supporting FHIR Search directly requires a feasibility executor (FLARE) to answer feasibility queries across multiple criteria. The advantage of the FLARE approach is that it did not only overcome current FHIR Search limitations but will also provide a solution to further limitations in the future. An example of this is the implementation of time-dependent intercriterion relationships (eg, a specific laboratory value within 3 days of a medication), which we plan to implement in the future. This is possible as full FHIR resources can be processed, including the appropriate time stamp field for each resource, which can then be compared for the specified interresource time constraints for each patient. Our performance analysis revealed that our implemented feasibility platform can answer queries for large data sets (multiple millions of resources) within seconds and that CQL is significantly faster than FLARE. The performance depends heavily on the number of patients for CQL and, for FLARE, the number of hits for each single criterion searched for. Consistent with this, we are planning to improve the performance of our implementation by using heuristics on the FHIR server to optimize FLARE and CQL query execution. This means identifying the criterion with the statistically lowest number of occurrences first, and then, querying further criteria with the reduced patient set. This is possible for FLARE and CQL and will be investigated by our team in the future. The implementation and design described here focused on the GECCO COVID-19 data set. The platform presented here is very generalizable and can be applied to any FHIR-formatted data or even to different query languages currently supported by different FHIR servers. One of the next steps is to integrate more data from different sources. In this pursuit, partners from the MII and German Biobank Alliance [57] have joined forces in 2021 to bring together previously independent initiatives for data and biosample sharing, by aligning information technology infrastructures and the respective regulatory and governance frameworks established in Germany within the biobanking community on one side and the medical informatics community on the other. The resulting Aligning Biobanks and DIC Efficiently [58] project started in May 2021 [59]. In our implementation, we only tested specific FHIR servers; however, our support of FHIR Search allows us to work with any standard FHIR API. Thus, testing our system with FHIR-APIs built on optimized database systems, as suggested by Paris et al [35], would be of interest. The platform presented here provides a solution only for the first part of the research cycle. Given the way the platform is built, currently, we return only the number of patients. One can easily imagine changing the return value to a list of patient IDs, which would allow the platform to create a cohort or patient subpopulation for a later decentral data selection process. This decentral cohort-creation process can then be combined with a decentral data selection process. This would allow a researcher to create a feature (criteria) set of data, based on the previously created cohort, which the researcher would like to extract for further analysis. Such a tool can then extract the required data and create a prepared data set for analysis at each site. In the simplest case, this prepared data set can be a comma-separated list of selected features for each patient. Creating such a tool would allow the FHIR standard to support distributed privacy-preserving analysis using tools such as DataSHIELD [54,60].

## Appendix

Multimedia Appendix 1 List of identified requirements for a feasibility platform.

Multimedia Appendix 2 Representation of a feasibility query as it moves through the system from user interface representation, via Structured Query to Clinical Quality Language and Fast Healthcare Interoperability Resources Search.

## Abbreviations

API: application programming interface
CODEX: COVID-19 data exchange
CQL: Clinical Quality Language
DIC: data integration center
DSF: data sharing framework
FDPG: Deutsches Forschungsdatenportal für Gesundheit
FHIR: Fast Healthcare Interoperability Resources
FLARE: Feasibility Analysis Request Executor
GECCO: German Corona Consensus Data Set
HiGHmed: Heidelberg-Göttingen-Hanover Medical Informatics
i2b2: Informatics for Integrating Biology and the Bedside
MII: Medical Informatics Initiative
MIRACUM: Medical Informatics in Research and Care in University Medicine
OMOP: Observational Medical Outcomes Partnership
PHT: Personal Health Train
REDCap: Research Electronic Data Capture
UI: user interface

## Source Code Availability

The source code of the project is available on GitHub [61].
