# Supplementary material for: The Architecture of a Feasibility Query Portal for Distributed COVID-19 Fast Healthcare Interoperability Resources (FHIR) Patient Data Repositories: Design and Implementation Study
Source: JMIR Med Inform. 2022 May 25;10(5):e36709. doi: 10.2196/36709 (PMC9135115; doi:10.2196/36709)
Supplement: Multimedia Appendix 1 [file medinform_v10i5e36709_app1.pdf]

Feature	Title	Description	Initial Prioritisation	Implemented within project	Reason for not implementing
1	Freetext search of criteria	It should be possible to search for criteria using a free text search.	medium	Yes	
2	Display results of freetext search - autosuggester + filter by ontology	Results of a freetext search should be displayed as the user types and it should be possible to filter the results by the main categories of the ontology.	medium	Yes	
3	Ontology-Tree search	It should be possible to search for criteria in an hierachical ontology tree based on clinical vocabulary.	high	Yes	
4	Ability to create Boolean expressions	It should be possible to combine criteria in logical boolean expressions using AND and OR.	high	Yes	
5	Negation of Boolean expressions on criterion level	Single Criteria should be negatable for each criterion.	high	No	Was implemented using the concept of Exclusion criteria instead, as this is the natural way to describe research studies. A negation of single criteria is then not neccessary. Further it is easier to implement in FHIR Search and potentially improves performance as the exclusion of a specific condition will return less results than the result of a :not filter.
6	Negation of Boolean expressions on group level	Single Criteria should be negatable on a group level.	high	Yes	
7	Context of the Boolean expression	The context of a query has to be considered. Potential contexts would be patient, encounter, or measurement-series.	high	Yes/No	Only the patient context was implemented, due to the complexity multiple contexts would create. Further contexts could be considered in future projects.
8	Check for the existence of a criterion (e.g. Diagnosis)	It should be possible to only check if a criterion exists for a patient without specifying a specific value.	high	Yes	
9	Filter criterion value - value set- small	Some criteria have values from a value set (e.g. "positiv" / "negativ" result for a test). These should be selectable.	high	Yes	
10	Filter criterion value - value set- large	Some criteria have values from larger value sets - like the bodysite of a Specimen. For this a search filter should be included in the user interface to make selecting value concepts easier.	low	No	We identified very few cirteria where this is relevant and therefore prioritised other features.
11	Filter criterion value - numeric	Numeric criteria like laboratory values should be supported.	high	Yes	
12	Filter criterion by time restriction	it should be possible to restrict the time within which a criterion lies.	high	No	Moved to to the next phase of the project given resource constraints.
13	Multiple filters on one criterion	Filters accross multiple fields of the same criterion (FHIR Resource) should be supported.	medium	No	Moved to to the next phase of the project given resource constraints. Further, most criteria do not require multiple filters.
14	Direct relationships between resources	It should be possible to put multiple criteria into a direct relationship. For example bio specimen for a particular diagnosis.	medium	No	Complexity of implementation and potential impact on performance. Further, the ability to implement this strongly depends on the data, which means that it might not be possible to implement this accross all criteria.
15	Functional (calculated) criteria	Support for functional criteria, which are calculated based on the data as the request is sent. For example: The age of a patient based on his birthdate at the time of execution.	low	No	Complexity of implementation as each functional criterion would have to be defined and implemented individually.
16	Simple and advanced search	It should be possible to switch between a simple and advanced search view to reduce the barriers of entry for new users.	low	No	Moved to next phase as our first prototype needed to be assessed and evaluated before developing this concept further. Additionally some of the more complex features were not implemented yet, which makes different search types less important.
17	Select a group of criteria, if of the same type	It should be supported to select a group of criteria, if they are of the same type. For example the selection of all types of diabetes.	medium	Yes	
18	Put groups of criteria into a time relationship	It should be possible to put criteria in a time relationship. For example criteria x and y happened within 3 month of criterion z.	low	No	Complexity of implementation, potential impact on performance.
