# Supplementary material for: The Architecture of a Feasibility Query Portal for Distributed COVID-19 Fast Healthcare Interoperability Resources (FHIR) Patient Data Repositories: Design and Implementation Study
Source: JMIR Med Inform. 2022 May 25;10(5):e36709. doi: 10.2196/36709 (PMC9135115; doi:10.2196/36709)
Supplement: Multimedia Appendix 2 [file medinform_v10i5e36709_app2.pdf]

UI Representation

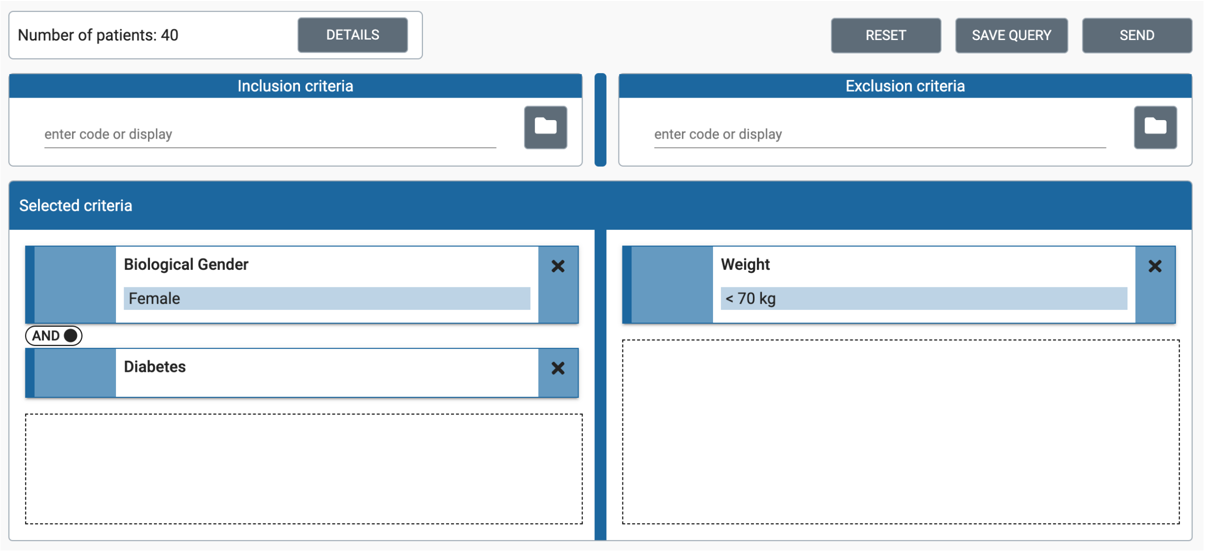

Structured-Query


```json
{
  "version": "http://to_be_decided.com/draft-1/schema#",
  "inclusionCriteria": [
    [
      {
        "termCodes": [
          {
            "code": "76689-9",
            "system": "http://loinc.org",
            "display": "Sex assigned at birth"
          }
        ],
        "valueFilter": {
          "type": "concept",
          "selectedConcepts": [
            {
              "code": "female",
              "system": "http://hl7.org/fhir/administrative-gender",
              "display": "Female"
            }
          ]
        }
      }
    ],
    [
      {
        "termCodes": [
          {
            "code": "E14",
            "system": "http://fhir.de/CodeSystem/bfarm/icd-10-gm",
            "display": "Nicht näher bezeichneter Diabetes mellitus"
          }
        ]
      }
    ]
  ],
  "exclusionCriteria": [
    [
      {
        "termCodes": [
          {
            "code": "27113001",
            "system": "http://snomed.info/sct",
            "display": "Body weight (observable entity)"
          }
        ],
        "valueFilter": {
          "type": "quantity-comparator",
          "comparator": "lt",
```

```
          "unit": {
            "code": "kg",
            "display": "kg"
          },
          "value": 70.0
        }
      }
    ]
  ]
}
```

CQL

```
library Retrieve
using FHIR version '4.0.0'
include FHIRHelpers version '4.0.0'

codesystem gender: 'http://hl7.org/fhir/administrative-gender'
codesystem icd10: 'http://fhir.de/CodeSystem/bfarm/icd-10-gm'
codesystem loinc: 'http://loinc.org'
codesystem snomed: 'http://snomed.info/sct'

context Patient

define Inclusion:
  exists from [Observation: Code '76689-9' from loinc] O
    where O.value.coding contains Code 'female' from gender and
  (
  exists [Condition: Code 'E14' from icd10] or
  exists [Condition: Code 'E14.3' from icd10] or
  exists [Condition: Code 'E14.31' from icd10] or
  exists [Condition: Code 'E14.30' from icd10] or
  exists [Condition: Code 'E14.1' from icd10] or
  exists [Condition: Code 'E14.11' from icd10] or
  exists [Condition: Code 'E14.0' from icd10] or
  exists [Condition: Code 'E14.01' from icd10] or
  exists [Condition: Code 'E14.2' from icd10] or
  exists [Condition: Code 'E14.21' from icd10] or
  exists [Condition: Code 'E14.20' from icd10] or
  exists [Condition: Code 'E14.7' from icd10] or
  exists [Condition: Code 'E14.75' from icd10] or
  exists [Condition: Code 'E14.74' from icd10] or
  exists [Condition: Code 'E14.73' from icd10] or
  exists [Condition: Code 'E14.72' from icd10] or
  exists [Condition: Code 'E14.4' from icd10] or
  exists [Condition: Code 'E14.41' from icd10] or
  exists [Condition: Code 'E14.40' from icd10] or
  exists [Condition: Code 'E14.8' from icd10] or
  exists [Condition: Code 'E14.81' from icd10] or
  exists [Condition: Code 'E14.80' from icd10] or
  exists [Condition: Code 'E14.5' from icd10] or
  exists [Condition: Code 'E14.51' from icd10] or
  exists [Condition: Code 'E14.50' from icd10] or
  exists [Condition: Code 'E14.6' from icd10] or
  exists [Condition: Code 'E14.61' from icd10] or
  exists [Condition: Code 'E14.60' from icd10] or
  exists [Condition: Code 'E14.9' from icd10] or
  exists [Condition: Code 'E14.91' from icd10] or
  exists [Condition: Code 'E14.90' from icd10])

define Exclusion:
  exists from [Observation: Code '27113001' from snomed] O
    where O.value as Quantity < 70.0 'kg'

define InInitialPopulation:
  Inclusion and
  not Exclusion
```

FHIR Search


```
[
  [
    [
      "http://localhost:8081/fhir/Observation?code=http%3A%2F%2Floinc.org%7C76689-9&value-concept=http%3A%2F%2Fhl7.org%2Ffhir%2Fadministrative-gender%7Cfemale"
    ],
    [
      "http://localhost:8081/fhir/Condition?code=http%3A%2F%2Ffhir.de%2FCodeSystem%2Fbfarm%2Ficd-10-gm%7CE14",
      "http://localhost:8081/fhir/Condition?code=http%3A%2F%2Ffhir.de%2FCodeSystem%2Fbfarm%2Ficd-10-gm%7CE14.3",
      "http://localhost:8081/fhir/Condition?code=http%3A%2F%2Ffhir.de%2FCodeSystem%2Fbfarm%2Ficd-10-gm%7CE14.1",
      "http://localhost:8081/fhir/Condition?code=http%3A%2F%2Ffhir.de%2FCodeSystem%2Fbfarm%2Ficd-10-gm%7CE14.0",
      "http://localhost:8081/fhir/Condition?code=http%3A%2F%2Ffhir.de%2FCodeSystem%2Fbfarm%2Ficd-10-gm%7CE14.2",
      "http://localhost:8081/fhir/Condition?code=http%3A%2F%2Ffhir.de%2FCodeSystem%2Fbfarm%2Ficd-10-gm%7CE14.7",
      "http://localhost:8081/fhir/Condition?code=http%3A%2F%2Ffhir.de%2FCodeSystem%2Fbfarm%2Ficd-10-gm%7CE14.4",
      "http://localhost:8081/fhir/Condition?code=http%3A%2F%2Ffhir.de%2FCodeSystem%2Fbfarm%2Ficd-10-gm%7CE14.8",
      "http://localhost:8081/fhir/Condition?code=http%3A%2F%2Ffhir.de%2FCodeSystem%2Fbfarm%2Ficd-10-gm%7CE14.5",
      "http://localhost:8081/fhir/Condition?code=http%3A%2F%2Ffhir.de%2FCodeSystem%2Fbfarm%2Ficd-10-gm%7CE14.6",
      "http://localhost:8081/fhir/Condition?code=http%3A%2F%2Ffhir.de%2FCodeSystem%2Fbfarm%2Ficd-10-gm%7CE14.9",
      "http://localhost:8081/fhir/Condition?code=http%3A%2F%2Ffhir.de%2FCodeSystem%2Fbfarm%2Ficd-10-gm%7CE14.31",
      "http://localhost:8081/fhir/Condition?code=http%3A%2F%2Ffhir.de%2FCodeSystem%2Fbfarm%2Ficd-10-gm%7CE14.30",
      "http://localhost:8081/fhir/Condition?code=http%3A%2F%2Ffhir.de%2FCodeSystem%2Fbfarm%2Ficd-10-gm%7CE14.11",
      "http://localhost:8081/fhir/Condition?code=http%3A%2F%2Ffhir.de%2FCodeSystem%2Fbfarm%2Ficd-10-gm%7CE14.01",
      "http://localhost:8081/fhir/Condition?code=http%3A%2F%2Ffhir.de%2FCodeSystem%2Fbfarm%2Ficd-10-gm%7CE14.21",
      "http://localhost:8081/fhir/Condition?code=http%3A%2F%2Ffhir.de%2FCodeSystem%2Fbfarm%2Ficd-10-gm%7CE14.20",
      "http://localhost:8081/fhir/Condition?code=http%3A%2F%2Ffhir.de%2FCodeSystem%2Fbfarm%2Ficd-10-gm%7CE14.75",
      "http://localhost:8081/fhir/Condition?code=http%3A%2F%2Ffhir.de%2FCodeSystem%2Fbfarm%2Ficd-10-gm%7CE14.74",
      "http://localhost:8081/fhir/Condition?code=http%3A%2F%2Ffhir.de%2FCodeSystem%2Fbfarm%2Ficd-10-gm%7CE14.73",
      "http://localhost:8081/fhir/Condition?code=http%3A%2F%2Ffhir.de%2FCodeSystem%2Fbfarm%2Ficd-10-gm%7CE14.72",
      "http://localhost:8081/fhir/Condition?code=http%3A%2F%2Ffhir.de%2FCodeSystem%2Fbfarm%2Ficd-10-gm%7CE14.41",
      "http://localhost:8081/fhir/Condition?code=http%3A%2F%2Ffhir.de%2FCodeSystem%2Fbfarm%2Ficd-10-gm%7CE14.40",
      "http://localhost:8081/fhir/Condition?code=http%3A%2F%2Ffhir.de%2FCodeSystem%2Fbfarm%2Ficd-10-gm%7CE14.81",
      "http://localhost:8081/fhir/Condition?code=http%3A%2F%2Ffhir.de%2FCodeSystem%2Fbfarm%2Ficd-10-gm%7CE14.80",
      "http://localhost:8081/fhir/Condition?code=http%3A%2F%2Ffhir.de%2FCodeSystem%2Fbfarm%2Ficd-10-gm%7CE14.51",
      "http://localhost:8081/fhir/Condition?code=http%3A%2F%2Ffhir.de%2FCodeSystem%2Fbfarm%2Ficd-10-gm%7CE14.50",
      "http://localhost:8081/fhir/Condition?code=http%3A%2F%2Ffhir.de%2FCodeSystem%2Fbfarm%2Ficd-10-gm%7CE14.61",
      "http://localhost:8081/fhir/Condition?code=http%3A%2F%2Ffhir.de%2FCodeSystem%2Fbfarm%2Ficd-10-gm%7CE14.60",
      "http://localhost:8081/fhir/Condition?code=http%3A%2F%2Ffhir.de%2FCodeSystem%2Fbfarm%2Ficd-10-gm%7CE14.91",
      "http://localhost:8081/fhir/Condition?code=http%3A%2F%2Ffhir.de%2FCodeSystem%2Fbfarm%2Ficd-10-gm%7CE14.90"
    ]
  ],
  [
    [
      "http://localhost:8081/fhir/Observation?code=http%3A%2F%2Fsnomed.info%2Fsct%7C27113001&value-quantity=lt70.0%7Ckg"
    ]
  ]
]
```
